# Glia of *C. elegans* coordinate a protective organismal heat shock response independent of the neuronal thermosensory circuit

**DOI:** 10.1126/sciadv.abq3970

**Published:** 2022-12-09

**Authors:** Holly K. Gildea, Phillip A. Frankino, Sarah U. Tronnes, Corinne L. Pender, Jenni Durieux, Julian G. Dishart, Hyun Ok Choi, Tayla D. Hunter, Shannon S. Cheung, Ashley E. Frakes, Edward Sukarto, Kevin Wickham, Andrew Dillin

**Affiliations:** ^1^ Howard Hughes Medical Institute, University of California, Berkeley, Berkeley, CA, USA.; ^2^ Helen Wills Neuroscience Institute, University of California, Berkeley, Berkeley, CA, USA.; ^3^ Department of Molecular and Cell Biology, University of California, Berkeley, Berkeley, CA, USA.; ^4^ Department of Biology, Howard University, Washington, DC, USA.; ^5^ National Institute of Diabetes and Digestive and Kidney Diseases, National Institutes of Health, Bethesda, MD, USA.

## Abstract

Aging organisms lose the ability to induce stress responses, becoming vulnerable to protein toxicity and tissue damage. Neurons can signal to peripheral tissues to induce protective organelle-specific stress responses. Recent work shows that glia can independently induce such responses. Here, we show that overexpression of heat shock factor 1 (*hsf-1*) in the four astrocyte-like cephalic sheath cells of *Caenorhabditis elegans* induces a non–cell-autonomous cytosolic unfolded protein response, also known as the heat shock response (HSR). These animals have increased lifespan and heat stress resistance and decreased protein aggregation. Glial HSR regulation is independent of canonical thermosensory circuitry and known neurotransmitters but requires the small clear vesicle release protein UNC-13. HSF-1 and the FOXO transcription factor DAF-16 are partially required in peripheral tissues for non–cell-autonomous HSR, longevity, and thermotolerance. Cephalic sheath glial *hsf-1* overexpression also leads to pathogen resistance, suggesting a role for this signaling pathway in immune function.

## Teaser

The cephalic sheath glia of the worm can coordinate a unique organismal heat shock response to increase longevity and health.

## INTRODUCTION

Cellular insults that can cause dysfunction occur as animals age. Cells have compartment-specific signaling pathways that detect such insults, temporarily limit protein production, and up-regulate protective genes, such as the protein folding assistant chaperones, to rescue cells from potentially toxic protein misfolding. As organisms experience damage over a lifetime, the cellular ability to mount responses to stress also declines (*1*, *2*). The process of aging perturbs cellular homeostasis by reducing organelle-specific unfolded protein response (UPR) induction and efficacy (*1*, *2*). Rescue of UPR functions by overexpression of activators in the nervous system increases healthspan and lifespan, indicating that UPRs are a potential therapeutic target for aging (*2*–*4*).

The compartment-specific UPR initiated by proteotoxic stress in the cytosol is known as the heat shock response (HSR) and is primarily mediated by the highly conserved transcription factor heat shock factor 1 (HSF-1) (*5*). Under nonstressed conditions, small heat shock protein chaperones such as HSP-70 and HSP-90 bind HSF-1, suppressing its activation (*6*). Upon detection of misfolded proteins in the cytosol, the chaperones are titrated away from HSF-1, freeing the transcription factor to trimerize and translocate into the nucleus (*6*). There, HSF-1 up-regulates chaperones and other genes that help resolve stress. HSF-1 activity declines with age, and this dysfunction occurs concomitant with worsening of cytosolic protein aggregation (*1*, *7*, *8*).

Recent work has established a unique role for the nervous system in initiating UPRs, including the HSR, across the whole organism (*2*–*4*, *9*–*11*). When the 302 neurons of *Caenorhabditis elegans* overexpress *hsf-1*, animals exhibit a non–cell-autonomous activation of the HSR in peripheral tissues, which leads to an increase in thermotolerance and lifespan (*3*). In *C. elegans*, heat sensing occurs via the canonical thermosensory circuit including AFD, AIY, and serotonergic neurons and is required for behaviors such as thermotaxis, although some other neurons can contribute to heat-related behaviors (*12*). Electrical activation of AFD sensory neurons and downstream ADF serotonergic neurons has been shown to induce peripheral HSF-1 activation in addition to canonical heat sensing behaviors, and this circuit has a demonstrated role in non–cell-autonomous HSR signaling (*3*, *11*, *13*). Thus, neural activity due to the sensory experience of heat, a potentially damaging insult, is coupled to the relevant organismal intracellular heat shock stress response.

Despite the neuronal ability to induce the HSR non–cell-autonomously when exogenously activated, neurons are likely not the most potent responders of the nervous system (*7*, *8*, *14*, *15*). Hyperthermia induces chaperone expression in neural cells; however, glia, particularly astrocytes, up-regulate chaperones more robustly than neurons do under these conditions (*14*, *15*). In vitro data suggest that glia may even provide chaperones to neurons directly (*16*). Neurons also aberrantly degrade HSF1 in several neurodegenerative disease conditions, including Alzheimer’s and Huntington’s diseases in model organisms and in human tissue (*7*, *8*). These findings suggest that glia, not neurons, are likely the primary coordinators of cytosolic stress responses in the nervous system.

*C. elegans* glia play an important role in the regulation of cellular stress and longevity (*17*, *18*). The 56 glia of *C. elegans* perform classic glial functions, supporting neuronal development, participating in synapses, and providing neurotransmitter and metabolic support to neurons (*19*, *20*). Four of these cells, the cephalic sheath (CEPsh) glia, most closely resemble mammalian astrocytes (*19*). CEPsh glia are poised at a unique junction of the environment, peripheral tissues, and the nervous system. They ensheath processes of sensory neurons that project their endings into the environment. These glia also surround the nerve ring, forming a barrier between the nerve ring and the rest of the body (*20*). Recent work has demonstrated that these cells are able to induce organelle-specific stress responses non–cell-autonomously in the case of the endoplasmic reticulum (ER) and mitochondria, but their role in cytosolic protein stress sensing and signaling has not been explored (*17*, *18*).

Here, we find that overexpression of *hsf-1* in the four CEPsh glia of *C. elegans* is able to coordinate an organismal HSR, confer stress resistance, and extend lifespan. Signaling of the glial HSR relies on a mechanism distinct both from that of neuronal HSR induction and from other glial stress responses. This response is independent of the canonical *C. elegans* neuronal thermosensory circuit for HSR induction and of dense core vesicle release. It requires the presence of small clear vesicle release machinery, although no single neurotransmitter known to be released through these vesicles is independently required for the peripheral HSR induction. CEPsh glial *hsf-1* coordinates the up-regulation of immune regulators, resulting in pathogen resistance. These data implicate *C. elegans* CEPsh glia as primary sensors and signalers of protein health insults, which can flexibly and specifically adopt signaling strategies to coordinate health and longevity across the organism.

## RESULTS

**Fig. 1.**
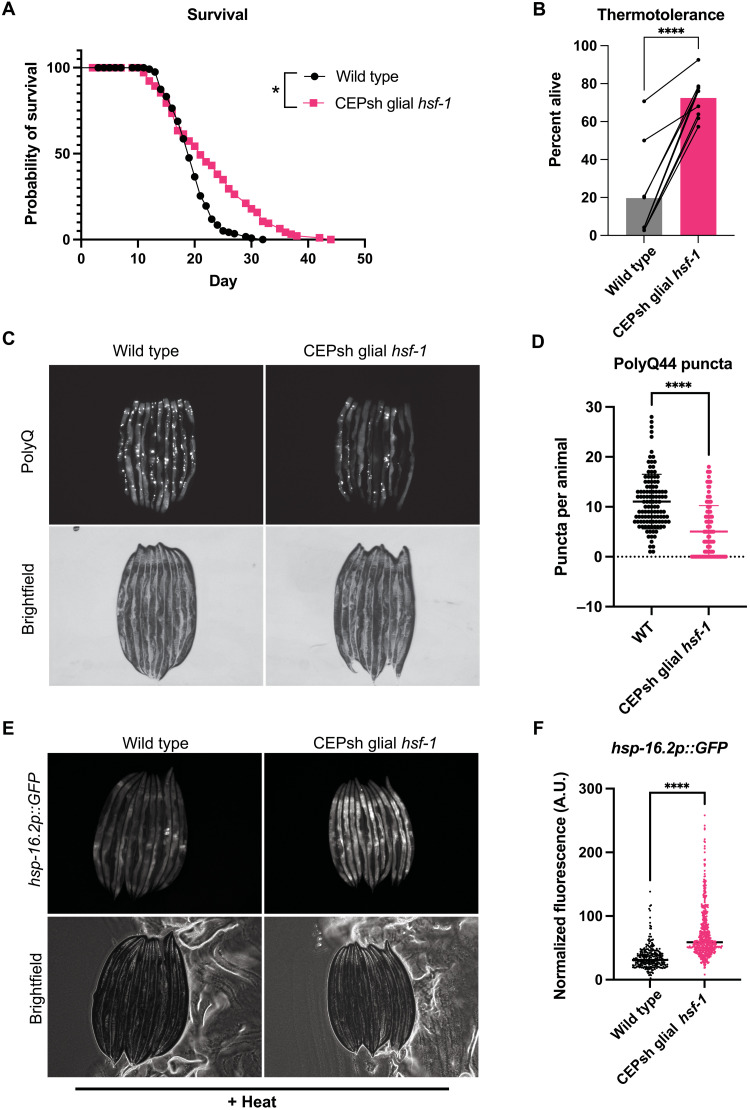
Overexpression of *hsf-1* in CEPsh glia increases life span and stress tolerance by nonautonomously inducing the HSR. (**A**) Survival of *hlh-17p::hsf-1* (integrated array) is significantly greater than that of wildtype N2 at 20°C. Mean survival of N2 = 19 days and *Is1(hlh-17p::hsf-1)* = 21 days, *P* = 0.017. (**B**) Thermotolerance of *Ex(hlh-17p::hsf-1)* array–expressing animals is significantly increased relative to wildtype N2 animals after heat stress at 34°C for 12 to 16 hours. Connected points are individual experiments, *P* < 0.0001. (**C**) Imaging of yellow fluorescent protein–tagged PolyQ44 fluorescence in wildtype versus *Ex(hlh-17p::hsf-1)* array–expressing animals, where puncta are aggregates of PolyQ44. (**D**) Quantification of representative experiment displayed in (C); *Ex(hlh-17p::hsf-1)* animals have significantly fewer puncta compared to wildtype (WT) PolyQ44 animals, *P* < 0.0001. (**E**) *hsp-16.2p::GFP* transcriptional reporter worms with and without *Ex(hlh-17p::hsf-1)* after mild heat stress and recovery, lined up head to tail. (**F**) Independent quantification of fluorescence by large particle flow cytometry via COPAS biosorter measurement. *Ex(hlh-17p::hsf-1)* worms (*N* = 507) are significantly brighter than *hsp-16.2p::GFP* alone (*N* = 290), *P* < 0.0001. Representative experiment shown of >3 experiments. A.U., arbitrary units. *P < 0.05, **P < 0.01, ***P < 0.001, and ****P < 0.0001.

To assess whether the four *C. elegans* CEPsh glia up-regulate a protective HSR organismally in response to *hsf-1*, we created strains overexpressing *hsf-1* under the CEPsh glia–specific promoter *hlh-17* (*hlh-17p::hsf-1*; strains listed in Table 1) (*17*, *21*, *22*). To evaluate the impact of *hlh-17p::hsf-1*, CEPsh glial *hsf-1*, on longevity, we first assayed lifespan under normal culture conditions. We found that CEPsh glial *hsf-1* animals were longer-lived than wildtype N2 animals (Fig. 1A and fig. S1, A and B). We also observed that this coincides with a suppression of fecundity (fig. S1, C and D). This is consistent with existing work suggesting that increased HSF-1 function in lifespan is a trade-off with reproductive fitness (*1*, *23*). This consequence may explain why *hsf-1* expression is tightly titrated across evolution. We next examined heat stress tolerance and found that CEPsh glial *hsf-1* animals are robustly thermotolerant compared to wildtype N2 animals (Fig. 1B and fig. S1E). We further observed that CEPsh glial *hsf-1* animals displayed lessened protein aggregation in a model expressing fluorescently tagged aggregation-prone polyglutamine compared to wildtype animals (Fig. 1, C and D). To test whether the increased health, longevity, and stress tolerance of CEPsh glial *hsf-1* animals correlate with organismal induction of HSR genes, we used a fluorescent transcriptional reporter for *hsp-16.2*, a heat shock chaperone induced upon heat stress. Using this system, we found that CEPsh glial *hsf-1* animals strongly up-regulated HSR chaperones and downstream genes upon heat stress compared to reporter animals alone, by imaging or by COPAS biosorter measurement, and that this increased expression was evident throughout the worm, predominantly visible in the intestine (Fig. 1, E and F, and fig. S1, F and G). Furthermore, CEPsh glial *hsf-1* animals failed to robustly up-regulate chaperone reporters associated with distinct stress responses, such as *hsp-4* and *hsp-6* (fig. S1H). We did not observe evidence of neuronal death in CEPsh glial *hsf-1* animals, although we saw mild morphology defects in the glia themselves (fig. S2, A and B). We also found that both the amphid sheath promoter *fig-1* and the pan-glial, though somewhat nonspecific promoter *mir-228* failed to induce a non–cell-autonomous HSR when overexpressing *hsf-1*, implying that there may be a dynamic interplay between multiple glial subtypes in which CEPsh glia are primarily activators of the HSR (fig. S2, C to E) (*24*). We thus determined that CEPsh glia nonautonomously induce the HSR, increasing longevity, stress response activation, and stress tolerance.

**Table 1.** Worm strain list.

Strain	Source	Identifier
*C. elegans*: strain N2 (Bristol)	CGC (Caenorhabditis Genetics Center)	N2
*C. elegans*: strain AGD2899, *uthls490[hlh-17p::hsf-1 FL::unc-54 3’UTR, myo-2p::tdTomato]*	This paper	AGD2899; in this paper, *Is1(hlh-17p::hsf-1)*
*C. elegans*: strain AGD2007, *uthEx861[hlh-17p::hsf-1 FL::unc-54 3′UTR, myo-2p::tdTomato]*	This paper	AGD2007; in this paper, *Ex(hlh-17p::hsf-1)*
*C. elegans*: strain AGD2008, *uthEx861[hlh-17p::hsf-1 FL::unc-54 3′UTR, myo-2p::tdTomato];dvln70[pCL25 (hsp-16.2p::GFP), pRF4(rol-6)]*	This paper	AGD2008
*C. elegans*: strain CL2070, *dvln70[pCL25 (hsp-16.2p::GFP), pRF4(rol-6)]*	CGC	CL2070
*C. elegans*: strain GF80, *dgEx80[pAMS66 vha-6p::Q44::YFP+rol-6(su1006)+pBluescriptII]*	CGC	GF80
*C. elegans*: strain AGD3598, *uthEx861[hlh-17p::hsf-1 FL::unc-54 3′UTR, myo-2p::tdTomato]; dgEx80[pAMS66 vha-6p::Q44::YFP + rol-6(su1006) + pBluescript II]*	This paper	AGD3598
*C. elegans*: strain FK134, *ttx-3(ks5) X*	CGC	FK134
*C. elegans:* strain AGD3250, *ttx-3(ks5) X; dvln70[pCL25 (hsp-16.2p::GFP), pRF4(rol-6)]*	This paper	AGD3250
*C. elegans*: strain AGD3055, *uthEx861[hlh-17p::hsf-1 FL::unc-54 3′UTR, myo-2p::tdTomato];dvln70[pCL25 (hsp-16.2p::GFP), pRF4(rol-6)]; ttx-3(ks-5)*	This paper	AGD3055
*C. elegans*: strain MT15434, *tph-1(mg280)II*	CGC	MT15434
*C. elegans*: strain AGD3254, *tph-1(mg280)II; dvln70[pCL25 (hsp-16.2p::GFP), pRF4(rol-6)]*	This paper	AGD3254
*C. elegans*: strain AGD3056, *uthEx861[hlh-17p::hsf-1 FL::unc-54 3′UTR, myo-2p::tdTomato]; dvln70[pCL25 (hsp-16.2p::GFP), pRF4(rol-6)]; tph-1(mg280)*	This paper	AGD3056
*C. elegans*: strain AGD1989, *unc-13(s69) I*, EG9631 backcrossed	CGC and Frakes *et al.* (*17*)	AGD1989
*C. elegans*: strain AGD3113, *dvln70[pCL25 (hsp-16.2p::GFP), pRF4(rol-6)]; unc-13(s69) I*	This paper	AGD3113
*C. elegans*: strain AGD3115, *uthEx861[hlh-17p::hsf-1 FL::unc-54 3′UTR, myo-2p::tdTomato]; dvln70[pCL25 (hsp-16.2p::GFP), pRF4(rol-6)]; unc-13(s69) I*	This paper	AGD3115
*C. elegans*: strain AGD3634, *uthEx861[hlh-17p::hsf-1 FL::unc-54 3′UTR, myo-2p::tdTomato]); unc-13(s69) I*	This paper	AGD3634
*C. elegans*: strain CB928, *unc-31(e928)IV*	CGC	CB928
*C. elegans*: strain AGD3054, *dvln70[pCL25 (hsp-16.2p::GFP), pRF4(rol-6)]; unc-31(e928)IV*	This paper	AGD3054
*C. elegans*: strain AGD3114, *uthEx861[hlh-17p::hsf-1 FL::unc-54 3′UTR, myo-2p::tdTomato]; dvln70[pCL25 (hsp-16.2p::GFP), pRF4(rol-6)]; unc-31(e928)IV*	This paper	AGD3114
*C. elegans*: strain AGD3029, *eat-4(ky5) III* backcrossed	CGC and the Dillin laboratory	AGD3029
*C. elegans*: strain AGD3268, *uthEx861[hlh-17p::hsf-1 FL::unc-54 3′UTR, myo-2p::tdTomato]; eat-4(ky5) III.*	This paper	AGD3268
*C. elegans*: strain AGD3038, *tdc-1(n3419) II* backcrossed	CGC and the Dillin laboratory	AGD3038
*C. elegans*: strain AGD3267, *uthEx861[hlh-17p::hsf-1 FL::unc-54 3′UTR, myo-2p::tdTomato]; tdc-1(n3419) II*	This paper	AGD3267
*C. elegans*: AGD3159, *unc-17(e245) IV* backcrossed	CGC and the Dillin laboratory	AGD3159
*C. elegans*: AGD3255, *unc-17(e245) IV; uthEx861[hlh-17p::hsf-1 FL::unc-54 3′UTR, myo-2p::tdTomato]*	This paper	AGD3255
*C. elegans*: strain MT15620, *cat-2(n4547) II*	CGC	MT15620
*C. elegans* strain AGD3253, *cat-2(n4547) II; uthEx861[hlh-17p::hsf-1 FL::unc-54 3′UTR, myo-2p::tdTomato]*	This paper	AGD3253
*C. elegans*: strain AGD3161, *unc-25(e156) III* backcrossed	CGC and the Dillin laboratory	AGD3161
*C. elegans*: AGD3201, *uthEx861[hlh-17p::hsf-1 FL::unc-54 3′UTR, myo-2p::tdTomato]; unc-25(e156) III*	This paper	AGD3201
*C. elegans*: strain AGD2281, *uthIs497[hlh-17p::hsf-1 FL::unc-54 3′UTR, myo-2p::tdTomato]*	This paper	AGD2281; in this paper, *Is2(hlh-17p::hsf-1)*
*C. elegans*: strain AGD2290, *rmIs223[pC12C8.1::GFP; rol-6(su1006) II]*, aka AM446 backcrossed	The Dillin laboratory and a gift from the Morimoto laboratory	AGD2290
*C. elegans*: strain AGD2302, *uthIs497[hlh-17p::hsf-1 FL::unc-54 3′UTR, myo-2p::tdTomato]; rmIs223[pC12C8.1::GFP; rol-6(su1006) II]*	This paper	AGD2302
*C. elegans*: strain AGD2293, *uthIs497[hlh-17p::hsf-1 FL::unc-54 3′UTR, myo-2p::tdTomato],dvln70[pCL25 (hsp-16.2p::GFP), pRF4(rol-6)]*	This paper	AGD2293
*C. elegans*: strain AGD2519, *uthEx927[fig-1p::hsf-1::unc-54 3′UTR, myo-2p::BFP]*	This paper	AGD2519
*C. elegans*: strain AGD2539, *uthEx927[fig-1p::hsf-1::unc-54 3′UTR, myo-2p::BFP]; dvln70[pCL25 (hsp-16.2p::GFP), pRF4(rol-6)]*	This paper	AGD2539
*C. elegans*: strain AGD2329, *uthEx904[mir-228p::hsf-1::unc-54 3′UTR, myo-2p::BFP]*	This paper	AGD2329
*C. elegans*: strain AGD2336, *uthEx904[mir-228p::hsf-1::unc-54 3′UTR, myo-2p::BFP]; dvln70[pCL25 (hsp-16.2 promoter::GFP transcriptional fusion), pRF4(rol-6)]*	This paper	AGD2336
*C. elegans*: strain AGD1570, *uthIs441(hlh-17p::GFP::unc-54); pRF4(rol-6)]*	Frakes *et al.* (*17*)	AGD1570
*C. elegans*: strain CL2166, *dvIs19[pAG15(gst-4p::GFP::NLS)] III*	CGC	CL2166
*C. elegans*: strain AGD2857, *uthIs497[hlh-17p::hsf-1 FL::unc-54 3′UTR, myo-2p::tdTomato]; dvIs19[pAF15(gst-4p::GFP::NLS)] III*	This paper	AGD2857

**Fig. 2.**
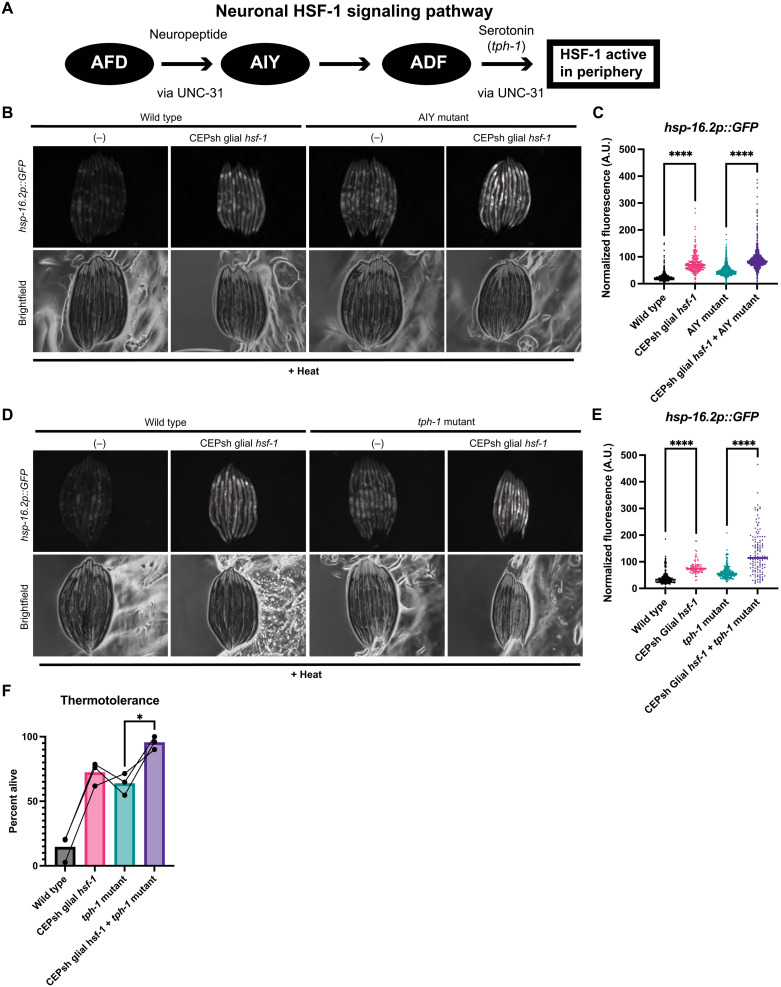
The canonical neuronal thermosensory HSR circuit is dispensable for CEPsh glial *hsf-1* signaling. (**A**) Schematic of the thermosensory circuit and relevant signaling components. (**B**) Mutants for AIY, *ttx-3(ks5)*, with and without *Ex(hlh-17p::hsf-1)* imaged for *hsp-16.2p::GFP* after mild heat stress and recovery. *Ex(hlh-17p::hsf-1); ttx-3(ks5)* exhibit higher *hsp-16.2p::GFP* fluorescence than *ttx-3(ks5)* alone. (**C**) Independent quantification of fluorescence by large particle flow cytometry via COPAS biosorter measurement. *Ex(hlh-17p::hsf-1); ttx-3(ks5)* are brighter than *ttx-3(ks5)* alone (*P* < 0.0001). wildtype *N* = 1014, CEPsh glial *hsf-1 N* = 253, *ttx-3 N* = 2767, and CEPsh glial *hsf-1 + ttx-3 N* = 1171. Representative experiment shown of three experiments. (**D**) Mutants for tryptophan hydroxylase, *tph-1(mg280)*, with and without *Ex(hlh-17p::hsf-1)* imaged for *hsp-16.2p::GFP* after mild heat stress and recovery. *Ex(hlh-17p::hsf-1); tph-1(mg280)* animals exhibit higher *hsp-16.2p::GFP* fluorescence than *tph-1(mg280)* animals alone. (**E**) Independent quantification of fluorescence by large particle flow cytometry via COPAS biosorter measurement. *Ex(hlh-17p::hsf-1); tph-1(mg280)* animals are significantly brighter than *tph-1(mg280)* alone (*P* < 0.0001). wildtype, *N* = 263; CEPsh glial *hsf-1*, *N* = 87; *tph-1*, *N* = 239; and CEPsh glial *hsf-1 + tph-1*, *N* = 131. Representative experiment shown of three experiments. (**F**) Thermotolerance of wildtype N2 animals, *Ex(hlh-17p::hsf-1)* animals, *tph-1(mg280)* animals, and *Ex(hlh-17p::hsf-1); tph-1(mg280)*. Data points are individual experiments, and connecting line indicates paired trials. Thermotolerance of *Ex(hlh-17p::hsf-1); tph-1(mg280)* is significantly greater than that of *tph-1(mg280)* (*P* = 0.04). *P < 0.05, **P < 0.01, ***P < 0.001, and ****P < 0.0001.

Under natural heat sensing conditions, the AFD thermosensory neuron signals to the AIY interneuron, which is upstream of serotonergic neurons such as NSM and ADF (Fig. 2A) (*11*, *25*). To ascertain whether the heat stress response to glial *hsf-1* is mediated by the canonical neuronal thermosensory circuitry for HSR induction, we measured the induction of *hsp-16.*2 in mutants defective in AIY interneuron formation, *ttx-3(ks5)*, with CEPsh glial *hsf-1* overexpression. *ttx-3(ks5)* mutants have been previously shown to decrease the induction of the HSR in otherwise wildtype animals (*9*). We found that under acute heat shock, *ttx-3(ks5)* mutant animals with CEPsh glial *hsf-1* still exhibited increased levels of *hsp-16.2* relative to *ttx-3(ks5)* mutants alone (Fig. 2, B and C). We thus determined that the AIY interneuron is not wholly required for the non–cell-autonomous signaling from CEPsh glia due to *hsf-1* overexpression. Unexpectedly, we also found that under this acute heat shock protocol, *ttx-3(ks5)* mutants may exhibit higher levels of *hsp-16.2p::GFP* relative to wildtype animals, although we did not consistently observe a significant increase by COPAS biosorter measurement across replicates. Together, these data suggest that the contribution of glia to HSR induction may be either downstream of or independent of AIY thermosensory neuron function, unlike the neuronal HSR.

Because of the apparent divergence of glial HSR regulation from this thermosensory circuit component, we next asked whether other members of the core HSR induction circuitry were dispensable for CEPsh glial *hsf-1* signaling. Serotonin and serotonin receptor activity are also required for downstream sensing of the AFD/AIY thermosensory circuit and HSR induction (*11*). Therefore, we next examined animals with the *tph-1(mg280)* mutation, which lack functional tryptophan hydroxylase and are unable to synthesize serotonin, for the induction of *hsp-16.2* by CEPsh glial *hsf-1*. We found that serotonin synthesis is dispensable for peripheral induction of *hsp-16.2* by CEPsh glial *hsf-1*, implying that glial HSR induction is independent of serotonin (Fig. 2, D and E). To further examine the role of serotonin, we subjected CEPsh glial *hsf-1* animals with and without the *tph-1(mg280)* mutation to chronic heat stress. We found that serotonin synthesis is not required for the glial *hsf-1*–mediated increase in thermotolerance (Fig. 2F). Unexpectedly, we also found that *tph-1(mg280)* animals may exhibit higher levels of *hsp-16.2p::GFP* and increased thermotolerance relative to wildtype animals in these assays. Together, the signaling of the HSR by CEPsh glia does not occur via the canonical neuronal heat stress pathway, nor does it require serotonin.

**Fig. 3.**
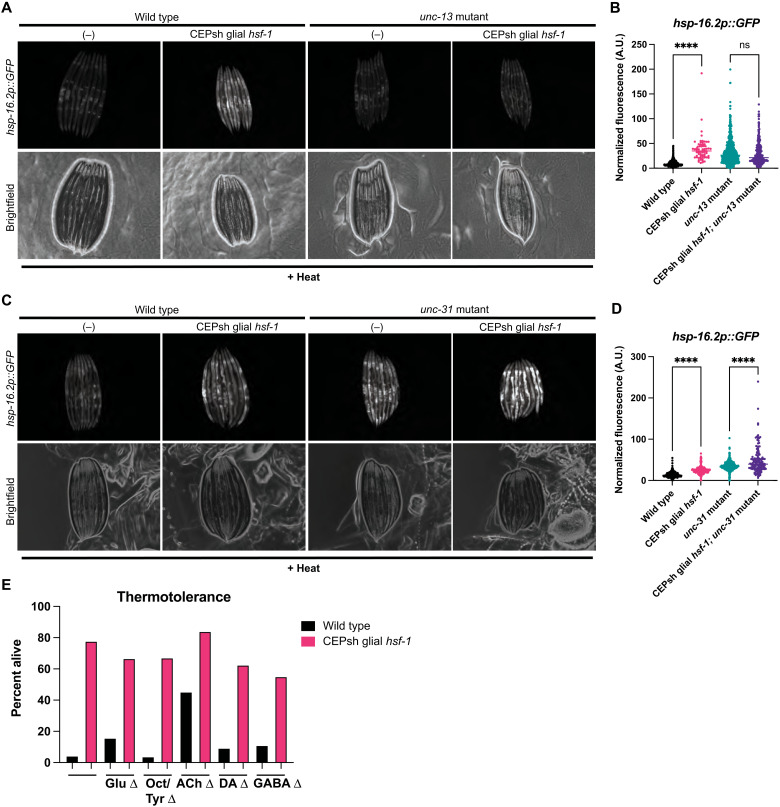
Small clear vesicles, but not dense core vesicles, are required for CEPsh glial *hsf-1* signaling via an unknown cargo. (**A**) Mutants for small clear vesicle release, *unc-13(s69)*, with and without *Ex(hlh-17p::hsf-1)* imaged for *hsp-16.2p::GFP* after mild heat stress and recovery. *Unc-13(s69)*; *Ex(hlh-17p::hsf-1)* animals have no increase in *hsp-16.2p::GFP*. (**B**) Independent quantification of fluorescence by large particle flow cytometry via COPAS biosorter measurement. Fluorescence of *Ex(hlh-17p::hsf-1); unc-13(s69); hsp-16.2p::GFP* animals is not significantly increased versus *unc-13(s69); hsp-16.2p::GFP*. wildtype, *N* = 548; CEPsh glial *hsf-1*, *N* = 67; *unc-13*, *N* = 1000; and CEPsh glial *hsf-1 + unc-13*, *N* = 304. Representative experiment shown of three experiments. ns, not significant. (**C**) Mutants for dense core vesicle release, *unc-31(e958)*, with and without *Ex(hlh-17p::hsf-1)* imaged for the reporter *hsp-16.2p::GFP* after mild heat stress and recovery. *unc-31(e958)*; *Ex(hlh-17p::hsf-1)* animals exhibit an increase in *hsp-16.2p::GFP*. (**D**) Independent quantification of fluorescence by large particle flow cytometry via COPAS biosorter measurement. Fluorescence of *Ex(hlh-17p::hsf-1); unc-31(e958); hsp-16.2p::GFP* animals is significantly increased relative to *unc-31(e958); hsp-16.2p::GFP* (*P* < 0.0001). wildtype, *N* = 559; CEPsh glial *hsf-1*, *N* = 166; *unc-31*, *N* = 535; and CEPsh glial *hsf-1 + unc-31*, *N* = 140. Representative experiment shown of three experiments. (**E**) Thermotolerance of *Ex(hlh-17p::hsf-1)* (pink) and wildtype N2 (black) animals. Neurotransmitter mutation is on the *x* axis, such that the leftmost comparison of *Ex(hlh-17p::hsf-1)* and wildtype N2 is in the wildtype background, followed by Glu = *eat-4(ky5)*, Oct/Tyr = *tdc-1(n3419)*, ACh = *unc-17(e245)*, DA = *cat-2(n4547)*, and GABA = *unc-25(e156)*. Representative experiment is displayed. *P < 0.05, **P < 0.01, ***P < 0.001, and ****P < 0.0001.

We next asked which signaling molecules might be responsible for this non–cell-autonomous signaling of the HSR to peripheral tissues by CEPsh glia, if not serotonin. Previous studies of stress signaling from glia in the case of the ER and mitochondrial UPRs implicated neuropeptides, although CEPsh glia could regulate distinct stress responses with similar or distinct signals (*17*, *18*). Dense core vesicles are required for the release of larger cargoes, such as neuropeptides, while small clear vesicles are required for neurotransmitter release (*26*, *27*). To determine whether small clear vesicles or dense core vesicles might be required for non–cell-autonomous HSR induction in CEPsh glial *hsf-1* animals, we used mutants for the vesicular release components *unc-13* and *unc-31*, respectively (*26*, *27*). We found that loss of small clear vesicle fusion via *unc-13(s69)* suppressed CEPsh glial *hsf-1* non–cell-autonomous induction of *hsp-16.2*; and we were unable to see a statistically significant preservation of thermotolerance increase (Fig. 3, A and B, and fig. S3A). In contrast, loss of dense core vesicle fusion via *unc-31(e958)* mutation failed to completely suppress the increase in peripheral *hsp-16.2* activation (Fig. 3, C and D) (*26*, *27*). Together, these data indicate that CEPsh glial signaling of the HSR relies on cargo enclosed in small clear vesicles and is independent of dense core vesicle neuropeptide signaling, unlike the neuronal and glial mitochondrial UPR and glial ER UPR responses (*17*, *18*).

As the canonical cargoes for small clear vesicles in the worm are neurotransmitters, we selected a set of mutants in synthesis or vesicular loading for each of the known neurotransmitters in *C. elegans*. Having already assessed serotonin, we turned our attention to mutants defective in signaling by glutamate (*eat-4*), γ-aminobutyric acid (GABA; *unc-25*), dopamine (*cat-2*), acetylcholine (*unc-17*), and octopamine and tyramine (*tdc-1*). Using the thermotolerance assay, we found that the increase in survival due to CEPsh glial *hsf-1* is preserved in the absence of dopamine, GABA, and octopamine/tyramine, and there is a trend toward significance in the survival increase of acetylcholine and glutamate mutants (Fig. 3E and fig. S3, B to F). Notably, acetylcholine and glutamate are canonical small clear vesicle cargoes, although the apparent partial suppression of effect suggests that neither transmitter is independently required for signaling, and we were able to observe increases in *hsp-16.2* by quantitative reverse transcription polymerase chain reaction (qRT-PCR) in both cases (fig. S3, A and B). These data suggest that no known neurotransmitter is independently responsible for organismal protection against heat stress conferred by CEPsh glial *hsf-1*, although the signal is likely contained in an UNC-13–mediated vesicle.

**Fig. 4.**
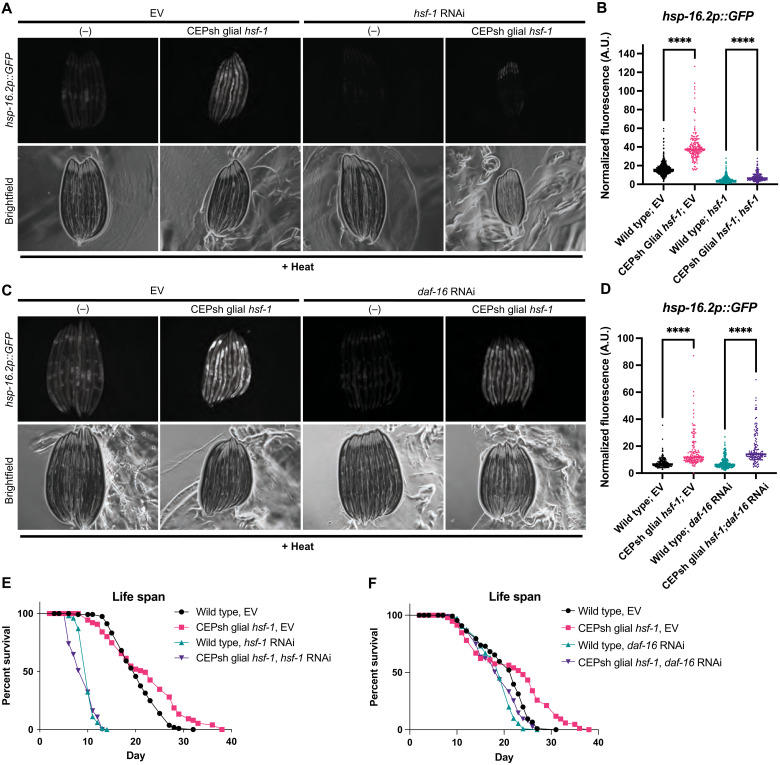
HSF-1 and DAF-16 are required for benefits of CEPsh glial *hsf-1* in peripheral tissues. (**A**) wildtype and *Ex(hlh-17p::hsf-1)* imaged for the reporter *hsp-16.2p::GFP* after mild heat stress and recovery under empty vector (EV) and *hsf-1* RNAi conditions. RNAi against *hsf-1* suppresses *hsp-16.2p::GFP*. (**B**) Independent quantification of fluorescence by large particle flow cytometry via COPAS biosorter measurement. *Ex(hlh-17p::hsf-1)*–related *hsp-16.2p::GFP* induction is partially suppressed by *hsf-1* RNAi, although *Ex(hlh-17p::hsf-1)* on *hsf-1* RNAi remains significantly increased versus wildtype N2 on *hsf-1* RNAi (*P* < 0.0001). wildtype on EV, *N* = 568; CEPsh glial *hsf-1* on EV, *N* = 169; wildtype on *hsf-1*, *N* = 707; and CEPsh glial *hsf-1* on *hsf-1*, *N* = 200. Representative experiment shown. (**C**) wildtype and *Ex(hlh-17p::hsf-1)* imaged for *hsp-16.2p::GFP* after mild heat stress and recovery on EV and *daf-16* RNAi. (**D**) Independent quantification of fluorescence by large particle flow cytometry via COPAS biosorter measurement. *Ex(hlh-17p::hsf-1)* on *daf-16* RNAi remains significantly increased versus wildtype N2 animals on *daf-16* RNAi (*P* < 0.0001). wildtype on EV, *N* = 165; CEPsh glial *hsf-1* on EV, *N* = 158; wildtype on *daf-16*, *N* = 272; and CEPsh glial *hsf-1* on *daf-16*, *N* = 139. Representative experiment shown. (**E**) lifespan of *Is1(hlh-17p::hsf-1)* versus wildtype N2 worms on EV and *hsf-1* bacteria. Mean survival of N2 on EV = 20 days, *Is1(hlh-17p::hsf-1)* on EV = 22 days, N2 on *hsf-1* RNAi = 10 days, and *Is1(hlh-17p::hsf-1)* on *hsf-1* RNAi = 10 days, *P* < 0.0001. (**F**) lifespan of *Is1(hlh-17p::hsf-1)* versus wildtype N2 worms on EV and *daf-16* bacteria. Mean survival of N2 on EV = 22 days, *Is1(hlh-17p::hsf-1)* on EV = 24 days, N2 on *daf-16* RNAi = 19 days, and *Is1(hlh-17p::hsf-1)* on *daf-16* RNAi = 19 days, *P* = 0.0012. *P < 0.05, **P < 0.01, ***P < 0.001, and ****P < 0.0001.

Neuronal HSR signaling relies on both HSF-1 and the insulin signaling-related FOXO homolog DAF-16 in peripheral tissues to enact survival benefits. Therefore, we tested the requirement for these transcription factors in peripheral HSR activation of CEPsh glial *hsf-1* animals (*3*). Neurons and CEPsh glia of *C. elegans* are partially resistant to RNA interference (RNAi), allowing us to interrogate peripheral signaling requirements specifically (fig. S4A) (*28*). Examining the induction of the *hsp-16.2* transcriptional reporter, we found that, as predicted, *hsf-1* is required in peripheral cells for most HSR chaperone induction (Fig. 4, A and B). In contrast, *daf-16* is not required for peripheral *hsp-16.2* induction, although we sometimes observed a small overall reduction in the level of *hsp-16.2* (Fig. 4, C and D). Despite these differences in chaperone induction, the increase in lifespan due to CEPsh glial *hsf-1* was largely dependent on both *hsf-1* and *daf-16* in peripheral tissues (Fig. 4, E and F). Furthermore, *hsf-1* and *daf-16* seem to be at least partially required for thermotolerance increase (fig. S4, B and C). Overall, these data indicate that *hsf-1* and *daf-16* act in concert to regulate the protective phenotypes of CEPsh glial *hsf-1* animals in the peripheral tissues.

**Fig. 5.**
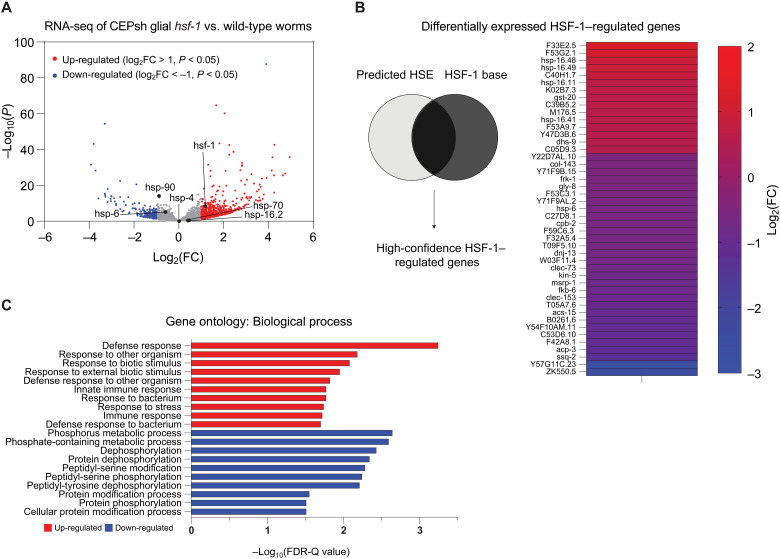
CEPsh glial *hsf-1* induces peripheral changes in HSF-1–regulated genes and the immune response. (**A**) Volcano plot demonstrating the magnitude [log_2_(FC)] and significance [−log_10_(*P* value)] of changes in gene expression from whole-animal RNA-seq of *Is1(hlh-17p::hsf-1)* versus wildtype N2. Labeled genes are stress genes, including *hsf-1* and HSR chaperones *hsp-70*, *hsp-16.2* (HSF-1 regulated), and *hsp-90* (non–HSF-1 regulated), as well as ER UPR chaperone *hsp-4* and mitochondrial UPR chaperone *hsp-6*. (**B**) High-confidence HSF-1–regulated genes are displayed alongside their log_2_(FC) values, color-coded from cool (down-regulated) to warm (up-regulated). (**C**) The top 10 GO terms for up-regulated (red) and down-regulated (blue) genes are displayed with their −log_10_-corrected false discovery rate (FDR)-Q value.

Beyond known HSR effectors, we next sought to identify organismal changes in gene expression that might shed light on the peripheral tissues’ interpretation of glial *hsf-1* signaling. Whole-animal RNA sequencing (RNA-seq) revealed substantial gene expression changes in CEPsh glial *hsf-1* animals compared to wildtype N2 animals, with 692 genes significantly up-regulated and 272 genes down-regulated [adjusted *P* ≤ 0.05 and log_2_(FC) (fold change) of greater than 1 or less than −1, respectively]. In CEPsh glial *hsf-1* animals, *hsf-1* is significantly up-regulated, and HSR genes *hsp-16.2* and *hsp-70* are mildly increased, while chaperones for the ER and mitochondrial UPRs were either unchanged or down-regulated, respectively (Fig. 5A). We further validated these transcriptional changes by the use of a fluorescent reporter for *gst-4*, a gene that exhibited a mild increase in expression in our dataset and by reporter imaging (fig. S5A). To identify high-confidence HSF-1–regulated genes that were differentially expressed in the CEPsh glial *hsf-1* animals, we generated a list of genes that were previously reported to be HSF-1 targets and had HSF-1 binding sites in the immediate upstream region from the start codon (*29*). Many high-confidence HSF-1 target genes were significantly up-regulated or down-regulated (*P* < 0.05) in CEPsh glial *hsf-1* animals (Fig. 5B). These data imply that HSF-1 may be activating as both a transcriptional activator and a repressor, as others have previously indicated (*29*–*31*). To evaluate the categories in which whole-animal gene expression was altered by sensing CEPsh glial *hsf-1*, we used gene ontology (GO) analysis of up-regulated and down-regulated genes (*32*, *33*). GO term enrichment analysis of the significantly up-regulated genes contained GO terms concerning the immune response and stress responses generally, while GO terms associated with the significantly down-regulated genes highlighted protein modification, specifically phosphorylation (Fig. 5C). These changes may reflect activation and/or inhibition of downstream signaling mechanisms. We also compared heat-stressed CEPsh glial *hsf-1* animals to heat-stressed wildtype animals via RNA-seq, observing a much smaller increase in HSR genes as wildtype animals themselves up-regulated the HSR in response to heat (fig. S5B). We observed a preservation of up-regulated GO terms related to the immune response in this comparison as well as down-regulated GO terms related to dephosphorylation and reproduction (fig. S5C). Overall, sequencing analysis of the CEPsh glial *hsf-1* animals reveals a broad up-regulation of immune and stress response genes with differential expression of many bona fide HSF-1 target genes.

**Fig. 6.**
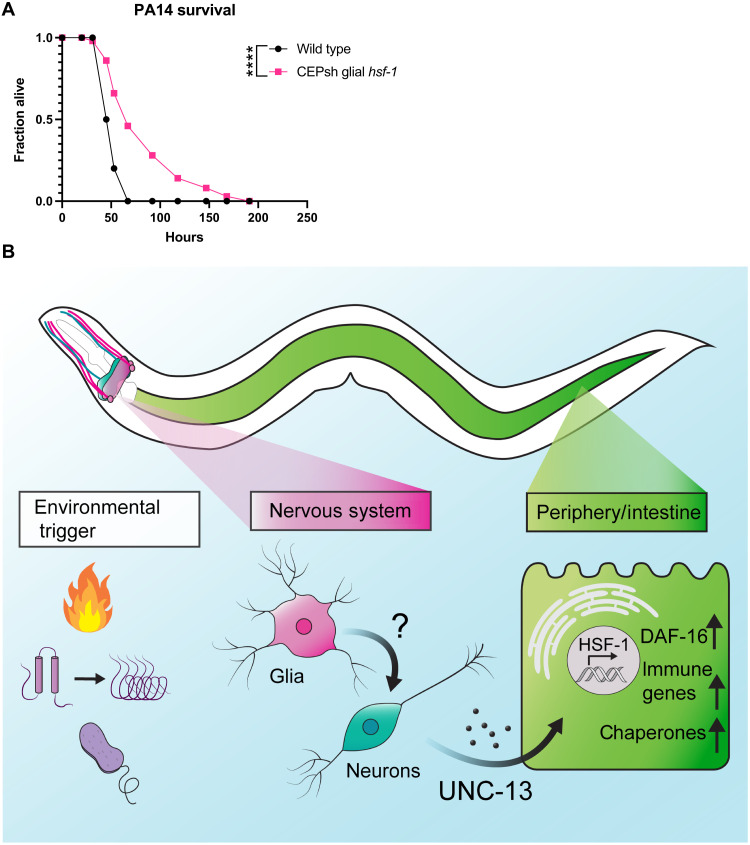
CEPsh glial *hsf-1* induces immune resistance. (**A**) Survival of *Ex(hlh-17p::hsf-1)* is increased versus wildtype N2 animals on *P. aeruginosa* bacteria. Mean survival for wildtype N2 animals = 53 hours and for *Ex(hlh-17p::hsf-1)* animals = 67 hours. *P* < 0.0001. (**B**) Schematic summarizing the findings. Heat, immune challenge, or misfolded proteins may activate signaling. Glial activation of *hsf-1* likely signals to neurons, causing the release of some *unc-13*–dependent cue to peripheral cells. Downstream cells activate HSF-1 and DAF-16 as well as immune factors to increase lifespan, stress tolerance, and immune resistance. *P < 0.05, **P < 0.01, ***P < 0.001, and ****P < 0.0001.

Infectious insults are important natural environmental stimuli for worms, and infection is a major cause of death across the organism’s lifespan (*34*). The bacteria *Pseudomonas aeruginosa* is pathogenic to *C. elegans*, and *hsf-1* is required for normal survival on *P. aeruginosa* (*35*). Furthermore, heat shock chaperones are activated upon exposure to the bacteria (*13*). Given data suggesting a broad up-regulation of immune genes in CEPsh glial *hsf-1* animals, we hypothesized that CEPsh glial *hsf-1* might induce pathogen resistance. We therefore tested the resistance of CEPsh glial *hsf-1* versus wildtype N2 worms on the *P. aeruginosa* PA14 strain using the slow killing assay and found a robust increase in survival in CEPsh glial *hsf-1* animals (Fig. 6A). These data suggest that CEPsh glial up-regulation of HSF-1 activity drives a true immune response that protects the animals from bacterial infection.

## DISCUSSION

We have identified a unique role for the four astrocyte-like CEPsh glia of *C. elegans* in coordinating a non–cell-autonomous HSR. Animals overexpressing *hsf-1* in CEPsh glia are more tolerant to heat stress, have decreased protein aggregation, and are longer-lived. These phenotypes correlate with an increase in HSR chaperones across the animal, demonstrating the ability of CEPsh cells to induce the stress response in distal tissues by a diffuse signaling mechanism (Fig. 6B).

*C. elegans* have 959 somatic cells, 302 neurons, and 56 glia, among which only 4 are CEPsh glia. Previous work demonstrated beneficial effects on longevity when overexpressing *hsf-1* in all 302 neurons, which amount to nearly one-third of the animal’s cells; in this study, however, we overexpress *hsf-1* in fewer than 0.5% of all cells of the worm and find similar effects on lifespan and stress tolerance (*3*). These data suggest that the worm is particularly responsive to stress signaling from CEPsh glia. As glia are best known for their interactions with neurons, this also suggests that glial stress responses may play a larger role in regulating neuronal stress response activity.

We show here that glial coordination of the HSR is independent of known components of the canonical thermosensory circuit for HSR induction. The AIY interneuron has been previously identified as a hub for integrating heat sensing (*3*, *9*); however, we find that this neuron is not required for non–cell-autonomous signaling of the CEPsh glial HSR. Our identification of an UNC-13–dependent, non-AIY-mediated glial signal for regulating lifespan via *hsf-1* signaling implies that two distinct pathways may be at work: one composed of thermosensory neuron components and the other downstream of CEPsh glia. This is further evident in the independence of glial HSR signaling from serotonin, a downstream component of the thermosensory circuit. The existence of two such pathways may explain the unexpected result that worms deficient in either AIY function or serotonin synthesis show a slight increase in heat shock chaperones upon acute heat shock relative to wildtype animals by reporter imaging in our hands. Lack of canonical signaling in the case of these mutants may cause compensatory up-regulation of the alternative pathway as a result, potentially via the CEPsh glia. Further work is needed to decipher the interaction between these systems.

Notably, the serotonin-independent nature of the CEPsh glial HSR mechanism differentiates it not only from the neuronal circuit controlling the HSR but also from all other known neuronally controlled proteostatic responses. Neurons ostensibly converge to regulate protein homeostasis using serotonin, which has been previously implicated in neuronal regulation of stress resistance via the HSR (*11*), the mitochondrial UPR (*36*), and the ER UPR (*37*). The CEPsh glial HSR mechanism is thus unique not only in the context of HSR regulation but also in the neural regulation of UPRs broadly. Furthermore, lifespan regulation by CEPsh glial signaling in the cases of the ER (*17*) and mitochondria (*18*) relies on neuropeptides, which are released via dense core vesicles, whereas the CEPsh glial HSR functions independent of dense core vesicle release. Although neuropeptides are certainly powerful diffusible cues, the HSR data suggest that release of neuropeptides is not the automatic glial response to stress across circumstances. Rather, the CEPsh glia are able to flexibly react to internal states to induce specific programs in distal cells. Thus, the CEPsh glial HSR is not a generalizable alarm system for any potential insult to the cells but rather specifically induces responses for relevant *hsf-1*–related stressors such as heat and immune challenge.

We find a requirement for the small clear vesicle release protein UNC-13 in the non–cell-autonomous communication of the HSR by CEPsh glia; however, the identity of the cue or cues contained in such vesicles remains unclear (Fig. 6B). As UNC-13 is thought to be expressed specifically in neurons, the most likely model involves CEPsh glial recruitment of neurons for signaling. We genetically disrupted the production or packaging of serotonin, dopamine, octopamine/tyramine, acetylcholine, GABA, and glutamate and failed to see a robust reduction in HSR signaling. Therefore, these signals could be functioning redundantly to induce the response, another unidentified cargo may be loaded into small clear vesicles, or glia may be modulating neurons in some other way, for example, by reuptake of neurotransmitters. First, a combination of transmitters may act to signal the glial HSR, potentially both acetylcholine and glutamate, for example. Also, distinct noncanonical neural cues may be at play. A unique small clear vesicle cargo derived from glia or from neurons may be responsible for this signaling. Several distinct stressors have been shown to induce lipids as a glial-neuronal stress signal, for example, which this work cannot rule out (*38*–*40*). CEPsh glia may also alter neurotransmitter release via damage signals, immune molecules, or even chaperones themselves, although these mechanisms are not well described in the glia of *C. elegans*. Last, CEPsh glia have been previously shown to alter neuronal activity via neurotransmitter reuptake, particularly in the case of glutamate (*41*). Unexpected baseline increases in thermotolerance and chaperone induction for several neurotransmitter mutants suggest that this is a likely mechanism, as decreases in synaptic levels of such a neurotransmitter could also be responsible for the changes of interest. CEPsh glial *hsf-1* may induce such modulation of neuronal activity via reuptake and could feasibly accomplish non–cell-autonomous HSR signaling through these means.

Despite substantial differences in initiation, both the glial and neuronal HSRs converge on the peripheral factors HSF-1 and DAF-16. The whole-animal RNA-seq data presented in this study suggest that *hsf-1* may be transcriptionally up-regulated in response to the glial HSR. Less unexpectedly, peripheral HSF-1 seems to be required for the induction of HSR chaperones, implying that activation of the transcription factor’s canonical activity in nonglial cells is necessary for the protein homeostasis effects of CEPsh glial *hsf-1*. The beneficial effects of glial *hsf-1* on lifespan are wholly dependent on *hsf-1*, in contrast to the neuronal *hsf-1* model in which HSF-1 is only partially required (*3*). These data suggest that HSF-1 may be an upstream component of the peripheral response, potentially able to activate other beneficial factors downstream. By contrast, the FOXO transcription factor DAF-16, which has been previously implicated in lifespan extension across perturbations including the neuronal HSR, is only partially required for glial *hsf-1* phenotypes (*3*). DAF-16 is canonically repressed by kinases downstream of the insulin receptor DAF-2 as part of the insulin and IGF-1 signaling pathway, and activation of DAF-16 is generally correlated with an increase in longevity (*42*–*44*). In the CEPsh glial *hsf-1* paradigm, DAF-16 is at least partially required for lifespan extension, thermotolerance, and, to a lesser extent, chaperone induction. However, in all cases, a slight increase remains despite peripheral knockdown of *daf-16*, supporting the hypothesis that DAF-16 may be downstream of HSF-1 or other induced protective factors (Fig. 6B).

We unbiasedly evaluated whole-animal gene expression by RNA-seq and found an unexpected enrichment of immune-related genes up-regulated in CEPsh glial *hsf-1* animals. HSF-1 has been previously implicated in immune function, and its role in pathogen resistance is independent of the canonical PMK-1/MAPK innate immune pathway, instead operating in a chaperone-dependent manner (*35*, *45*). In the worm, infection is a major cause of death, detectable by pharyngeal swelling, and *hsf-1* knockdown increases pharynx bacterial colonization (*34*, *46*). Data here indicate that CEPsh glia are able to induce a proimmune and prolongevity program by activating *hsf-1*, possibly increasing cellular protection from pathogens via induction of chaperones and immune response genes. By activating HSF-1–related genes specifically in this paradigm, we were able to achieve an effective increase in immune function in adult animals without a deleterious effect of prolonged immune activation on longevity. If increased HSF-1 function can protect cells from both proteotoxicity and pathogenic insults, we would anticipate that its activity would be positively selected evolutionarily. However, the negative impact of *hsf-1* up-regulation on reproductive function as demonstrated here suggests that evolutionary titration of function may balance these phenotypes to preserve the health of parents and offspring.

CEPsh glia are well positioned to receive cues from the environment, neurons, and peripheral tissues. This study, along with those detailing the role of these cells in the ER and mitochondrial UPRs, suggests that these cells may act as sensory organs particularly for organismal insults, inducing relevant and specific stress responses across the whole animal (*17*, *18*). The worm has no circulating adaptive immune system; however, the nervous system of *C. elegans* serves as an immune effector, regulating responses to toxic stimuli in coordinated behavioral and cellular programs. The connection between nervous system function and immune signaling in this case points to the larger role of the nervous system itself as the prototype for adaptive immunity.

CEPsh glia are thus able to coordinate multiple protective functions by non–cell-autonomous communication of the HSR. Considering the aging-related decline of function in the neuronal HSR and its relationship to protein aggregation, manipulation of glial *hsf-1* emerges as a promising tool to tackle aging and neurodegenerative phenotypes broadly.

## MATERIALS AND METHODS

### Thermotolerance

Worms were synchronized by bleaching as described here, L1 arrested, and plated on HT115 bacteria. At late D1, 15 worms per plate with five plates per condition were exposed to 34°C heat via an incubator for 13 to 16 hours. Plates were then removed from the incubator and manually assessed for movement and pharyngeal pumping, using light head taps where necessary, to determine survival. Worms that displayed internal hatching or crawled onto the side of the plate and desiccated were censored and omitted from the final analysis, and censorship criteria were predetermined before experimentation. Percent alive was calculated using the number of living worms divided by the total number of worms less censored worms for each strain. All experiments were performed a minimum of three independent times, except for *unc-25* thermotolerance, which was performed twice. Experiments were performed blinded in all cases. Displayed points represent independent experiments.

### Lifespan

Lifespan experiments were performed as previously described (*17*). In brief, worms were synchronized by bleaching, L1 arrested, and plated on HT115 bacteria. On day 1 of adulthood, worms were moved to fresh plates with 15 worms per plate and 10 plates per condition. Living worms were counted every day and occasionally every other day for the duration of the lifespan. Life was assessed by movement, pharyngeal pumping, or response to a light head touch. Worms were censored if they crawled onto the side of the plate and desiccated, if they displayed internal hatching, or if they had extruded vulvas/intestines, and censorship criteria were predetermined before experimentation. All lifespan experiments were performed a minimum of three times, except for the *mir-228* lifespan experiment in fig. S2, which was performed twice. Lifespan experiments were blinded in all cases. Representative experiments are displayed.

### *P. aeruginosa* survival

PA14 bacteria were cultured overnight at 37°C and protected from light in King's B (KB) media. A total of 20 μl was spread onto slow killing plates, which were incubated for 24 hours at 37°C and protected from light. After plates returned to room temperature, synchronized L4 worms were added to the plates, using six plates of 20 worms per plate. Survival was assessed as described above. Missing worms and those crawling onto the side of the plate were censored and omitted from analysis, but bagged worms were counted as dead for this assay, and censorship criteria were predetermined before experimentation. Worms were counted at least once per day but more frequently near peak death. Experiments were performed blinded in all cases and performed at least three independent times. Statistics were performed as described for lifespan experiments.

### Imaging

For normal fluorescent imaging, worms were anesthetized using 100 μM sodium azide solution on Nematode Growth Medium (NGM) plates, immediately aligned with a worm pick head to tail and imaged. Fluorescent and bright-field images were collected via the Echo Revolve Microscope. Exposure time and laser intensity were matched within each experiment. For experiments showing extrachromosomal arrays, animals were selected on the basis of the presence of a red coinjection marker while blinded to their green fluorescence via the NIGHTSEA benchtop fluorescence adapter for red fluorescence only. Integrated strain worms were picked for imaging on a nonfluorescent stereoscope to remain blinded to green fluorescence. All imaging experiments were performed at least three independent times.

### Dye filling

Worms were synchronized as described. At day 1, worms were washed off plates using M9. Leaving 1 ml of M9, we added 5 μl of DiO (Thermo Fisher Scientific, D3898) to the solution and rotated at 20°C for 3 hours. Worms were then washed with M9, plated on OP50, and left to recover overnight. Worms were anesthetized using 100 μM sodium azide on glass slides with agar pads and imaged using low laser power to avoid bleed-through of the *tdtomato* coinjection marker in CEPsh glial *hsf-1* animals. Images were acquired using the Zeiss Axio Observer Microscope with AiryScan. For postprocessing, Fiji was used to create maximum-intensity projections, which are displayed. Experiment was performed at least three times.

### Worm growth and maintenance

Worms were maintained at 15°C on NGM plates spotted with 200 μl of OP50 bacteria. Worms were chunked or picked for experiments onto NGM plates with 1 ml of OP50 and grown at 20°C. They were then synchronized for experiments as described here.

### Synchronization

Worms were synchronized by bleaching as previously described (*47*). In brief, worms were collected off plates into 15-ml conical tubes using M9 solution. Bleach solution was added until animals dissolved, and the worms were spun down (30 s at 1000 RCF) and washed five or more times with M9 before L1 arrest. L1 arrest was performed by suspending worms in M9 in 15-ml conical tubes and rotating overnight at 20°C before plating on OP50 or HT115 bacteria.

### RNAi feeding

RNAi feeding was performed as previously described (*3*, *17*).

### RNA isolation, library preparation, and sequencing

Animals were bleach synchronized and grown to the L4 stage on HT115 plates. At least 2000 animals per condition per replicate were washed off plates using M9 and collected. After a 30-s spin at 1000 RCF, M9 was aspirated and replaced with 1 ml of TRIzol, and the tube was immediately frozen in liquid nitrogen to be stored at −80°C for downstream processing. RNA was harvested after three freeze-thaw cycles in liquid nitrogen/37°C water bath. After the final thaw, 200 μl (1:5 chloroform:TRIzol) of chloroform solution was added to the sample and vortexed, and the aqueous phase was collected after centrifugation in a gel phase lock tube. RNA was isolated from the obtained aqueous phase using a Qiagen RNeasy Mini Kit according to the manufacturer’s directions. Library preparation was performed by Azenta Genewiz as follows: Extracted RNA samples were quantified using a Qubit 2.0 Fluorometer (Life Technologies, Carlsbad, CA, USA), and RNA integrity was checked using Agilent TapeStation 4200 (Agilent Technologies, Palo Alto, CA, USA). RNA-seq libraries were prepared using the NEBNext Ultra RNA Library Prep Kit for Illumina following the manufacturer’s instructions (NEB, Ipswich, MA, USA). Briefly, mRNAs were first enriched with oligo(dT) beads. Enriched mRNAs were fragmented for 15 min at 94°C. First- and second-strand complementary DNAs (cDNAs) were subsequently synthesized. cDNA fragments were end-repaired and adenylated at 3′ ends, and universal adapters were ligated to cDNA fragments, followed by index addition and library enrichment by limited-cycle PCR. The sequencing libraries were validated on the Agilent TapeStation (Agilent Technologies, Palo Alto, CA, USA) and quantified by using a Qubit 2.0 Fluorometer (Invitrogen, Carlsbad, CA) and by qPCR (KAPA Biosystems, Wilmington, MA, USA). The sequencing libraries were clustered on one lane of a flow cell. After clustering, the flow cell was loaded on the Illumina HiSeq instrument (4000 or equivalent) according to the manufacturer’s instructions. The samples were sequenced using a 2 × 150 base pair (bp) paired-end configuration. Image analysis and base calling were conducted by the HiSeq Control Software. Raw sequence data (.bcl files) generated from Illumina HiSeq were converted into fastq files and demultiplexed using Illumina’s bcl2fastq 2.17 software. One mismatch was allowed for index sequence identification.

### RNA-seq analysis

For RNA-seq analysis, the sequencing data were uploaded to the Galaxy project web platform, and the public server at usegalaxy.org was used to analyze the data (*48*). Paired-end reads were aligned using the Kallisto quant tool (version 0.46.0) with WBcel235 as the reference genome. FC values and statistics were generated using the DESeq2 tool with Kallisto quant count files as the input. Volcano plots were generated using GraphPad Prism software [version 9.2.0 (283)] on the FC and adjusted *P* values generated by the previous analysis. GO terms for differentially expressed genes were analyzed by using the GOrilla tool (http://cbl-gorilla.cs.technion.ac.il/#ref) on lists of genes that were up- or down-regulated (log_2_FC > 1 and log_2_FC < 1, respectively) with an adjusted *P* ≤ 0.05 (*32*, *33*). The raw RNA-seq data were uploaded to the National Center for Biotechnology Information Sequence Read Archive (PRJNA801195). Access is available at www.ncbi.nlm.nih.gov/bioproject/PRJNA801195.

### Quantitative reverse transcription polymerase chain reaction

RNA was isolated from day 1 adult animals as described above. In the case of array animals, red heads were manually enriched by picking onto plates and then isolated. cDNA was synthesized using the Qiagen RT kit and PCR run on QuantStudio using SYBR Green. Analysis was performed using the delta delta Ct method using the housekeeping genes *cdc-42*, *pmp-3*, and *y45F10D.4* unless otherwise noted. qPCR primers are listed as follows: *cdc-42*: AGG AAC GTC TTC CTT GTC TCC (forward) and GGA CAT AGA AAG AAA AAC ACA GTC AC (reverse); *pmp-3*: CGG TGT TAA AAC TCA CTG GAG A (forward) and TCG TGA AGT TCC ATA ACA CGA (reverse); *Y45F10D.4*: AAG CGT CGG AAC AGG AAT C (forward) and TTT TTC CGT TAT CGT CGA CTC (reverse); *hsp-16.2*: TCC ATC TGA GTC TTC TGA GAT TGT TA (forward) and TGG TTT AAA CTG TGA GAC GTT GA (reverse).

### Generation and integration of arrays

The *hlh-17* promoter was cloned into a vector containing full-length *hsf-1*, with sequences as previously described (*3*, *17*). Wildtype N2 strain worms were injected with the *hlh17p::hsf-*1; *unc-54* 3′UTR (untranslated region) plasmid and the *myo-2p::tdtomato* coinjection marker. Integration of extrachromosomal array lines was performed by γ-irradiation [*Is2(hlh-17p::hsf-1)*] or by ultraviolet irradiation [*Is1(hlh-17p::hsf-1)*]. Integrated lines were then backcrossed at least eight times to the wildtype N2 strain. Because of rapid transgene suppression of the integrated strains, the extrachromosomal array was used in all cases involving crosses, except for data in figs. S1 (G and H) and S5. Integrated strains were also used for all lifespans (except for fig. S1B) and for thermotolerance data in fig. S1E, as well as for RNA-seq.

### Brood size

Synchronized L4 animals were picked individually onto fresh HT115 bacteria plates and allowed to lay eggs for 24 hours at 20°C. They were then moved to fresh plates for each consecutive 24-hour period for the duration of the reproductive lifespan for at least 5 days. Progeny plates were allowed to grow up at 20°C for 2 days, and surviving larvae were imaged using the MBF Bioscience WormLab imaging system and counted. Experiments were performed at least three independent times.

### Heat shock for imaging

Synchronized worms were placed in a 34°C incubator for 2 hours, followed by a recovery for 2 hours at 20°C, at which time worms were imaged or biosorted as described.

### Heat shock for RNA-seq

Synchronized worms were placed in a 34°C incubator for 30 min, at which time the worms were collected in TRIzol as described above.

### Prediction of HSF-1 binding sites in *C. elegans* promoters

HSF-1 binding sites were predicted in the upstream regions of coding sequences using the FIMO tool (version 5.0.5) on MEME Suite (*49*, *50*). Briefly, 500-bp upstream flanks of all annotated coding genes were downloaded from the WormBase ParaSite to represent putative promoter regions (*51*). The HSF-1 position weight matrix (PWM) was downloaded from JASPAR (matrix ID MA0486.2) (*52*). FIMO was run with the HSF-1 PWM as input motif and the putative promoter regions as input sequences with a match *P* value < 1 × 10^−5^ to find 646 genes with HSF-1 binding sites. GO term analysis using the GOrilla tool confirmed the top GO terms of these genes to include chaperone-mediated folding (GO:0061077), protein folding (GO:0006457), and response to heat (GO:009408).

### COPAS biosorting and analysis

Worm sorting using the COPAS biosorter (Union Biometrica) was performed as previously described (*47*). In brief, worms were heat-shocked as described for *hsp-16.2p::GFP* conditions. Then, they were washed off plates into the sample cup using M9 and sorted. Laser photomultiplier tube values were consistent within experiments. All raw data were saved. For analysis, reads with time of flight (TOF) greater than 100 and extension (EXT) greater than 50 were included, and reads with lower values were excluded. Reads for which EXT or green peak height reached the maximum saturated value for the instrument of 65,532 were excluded. Normalized fluorescence was calculated by dividing the green peak height by TOF. For red-headed animals, worms with a red peak height of 1000 or greater were included, and lower values were presumed extrachromosomal array negative and were excluded. All sorting experiments were performed at least three independent times.

### PolyQ preparation and puncta quantification

Animals were age-synchronized by picking L4-staged animals derived from timed egg lays on NGM plates spotted with OP50. Animals were manually moved at day 2 of adulthood to new plates, away from their progeny. Fluorescent microscopy was performed at day 3 of adulthood animals. Fluorescent images were blinded, and yellow fluorescent protein–positive puncta were counted per animal for a minimum of 100 animals. Experiment was repeated three independent times.

### Genetic crosses

Males were generated either by heat exposure or by crossing to wildtype males. Hermaphrodites and males of interest were placed on NGM plates with a small amount of OP50 bacteria and allowed to mate. Progeny were singled onto individual plates for the F_1_ and the subsequent F_2_ generation and were screened for relevant phenotypes.

### Statistical analyses

Statistical analysis was performed using GraphPad Prism 9.2.0 (283), except for RNA-seq analysis, which was performed as described above. Individual analyses are as described in the figure legends. Lifespans were analyzed by the Gehan-Breslow-Wilcoxon test. Two condition comparisons were otherwise analyzed by two-tailed *t* test, with Welch’s correction where applicable, and more than two condition comparisons were analyzed by one-way analysis of variance (ANOVA) with Sidak’s multiple comparisons. The brood size assay experiment was analyzed via a Kolmogorov-Smirnov test because of our inability to assume a Gaussian distribution.

## Notes

View/request a protocol for this paper from *Bio-protocol*.
