# Supplementary material for: Glia of C. elegans coordinate a protective organismal heat shock response independent of the neuronal thermosensory circuit
Source: Sci Adv. 2022 Dec 9;8(49):eabq3970. doi: 10.1126/sciadv.abq3970 (PMC9733925; doi:10.1126/sciadv.abq3970)
Supplement: Supplementary file 1 — Figs. S1 to S5 [file sciadv.abq3970_sm.pdf]

Science Advances
AAAS

Supplementary Materials for

Glia of *C. elegans* coordinate a protective organismal heat shock response independent of the neuronal thermosensory circuit

Holly K. Gildea *et al.*

Corresponding author: Andrew Dillin, dillin@berkeley.edu

Sci. Adv. **8**, eabq3970 (2022)
DOI: 10.1126/sciadv.abq3970

This PDF file includes:

Figs. S1 to S5

S1

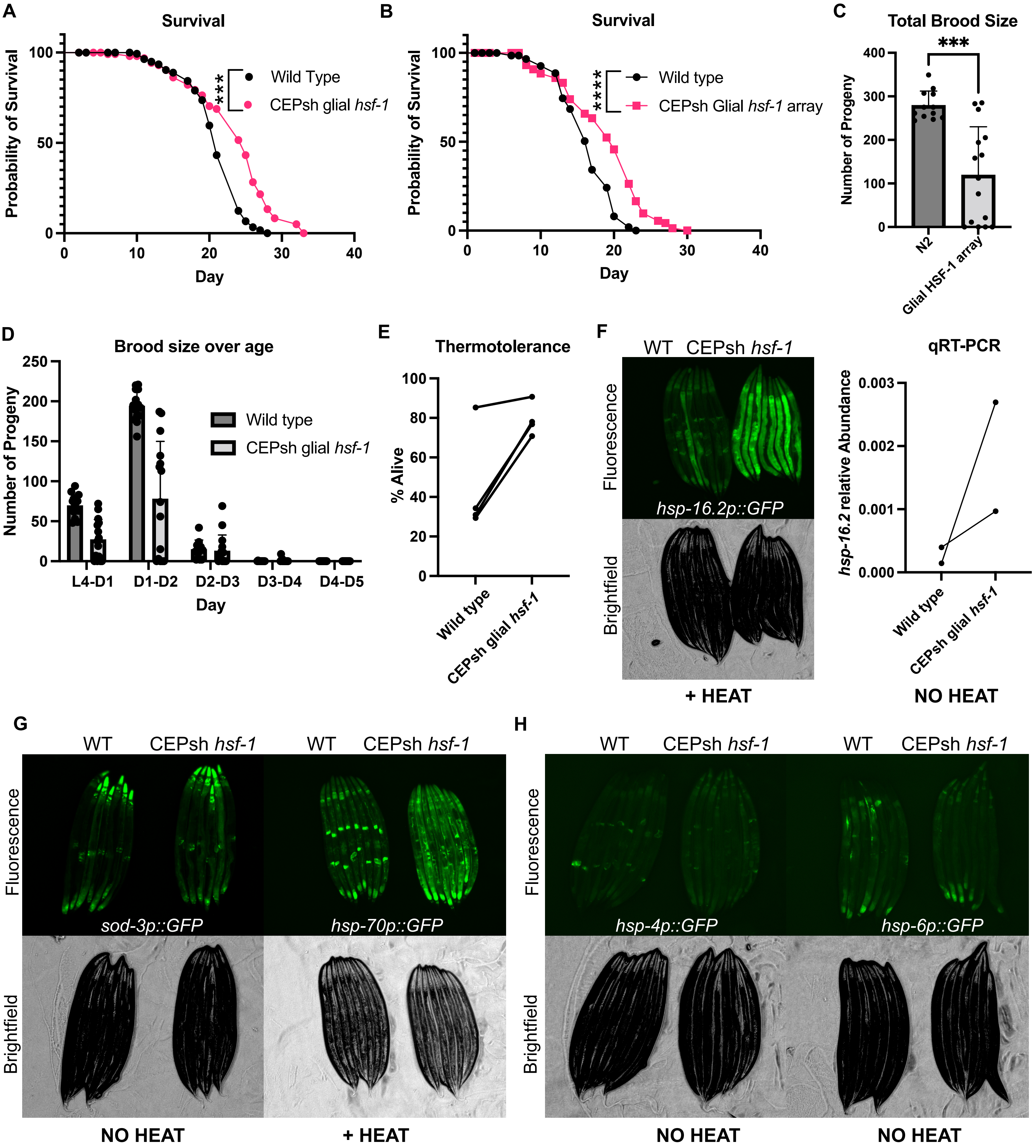
A
Survival
Probability of Survival
Day
Wild Type
CEPsh glial hsf-1
***
B
Survival
Probability of Survival
Day
Wild type
CEPsh Glial hsf-1 array
****
C
Total Brood Size
Number of Progeny
N2
Glial HSF-1 array
***
D
Brood size over age
Number of Progeny
Wild type
CEPsh glial hsf-1
L4-D1
D1-D2
D2-D3
D3-D4
D4-D5
Day
E
Thermotolerance
% Alive
Wild type
CEPsh glial hsf-1
F
WT CEPsh hsf-1
Fluorescence
Brightfield
hsp-16.2p::GFP
+ HEAT
qRT-PCR
hsp-16.2 relative Abundance
Wild type
CEPsh glial hsf-1
NO HEAT
G
WT CEPsh hsf-1
WT CEPsh hsf-1
Fluorescence
Brightfield
sod-3p::GFP
hsp-70p::GFP
NO HEAT
+ HEAT
H
WT CEPsh hsf-1
WT CEPsh hsf-1
Fluorescence
Brightfield
hsp-4p::GFP
hsp-6p::GFP
NO HEAT
NO HEAT

S2

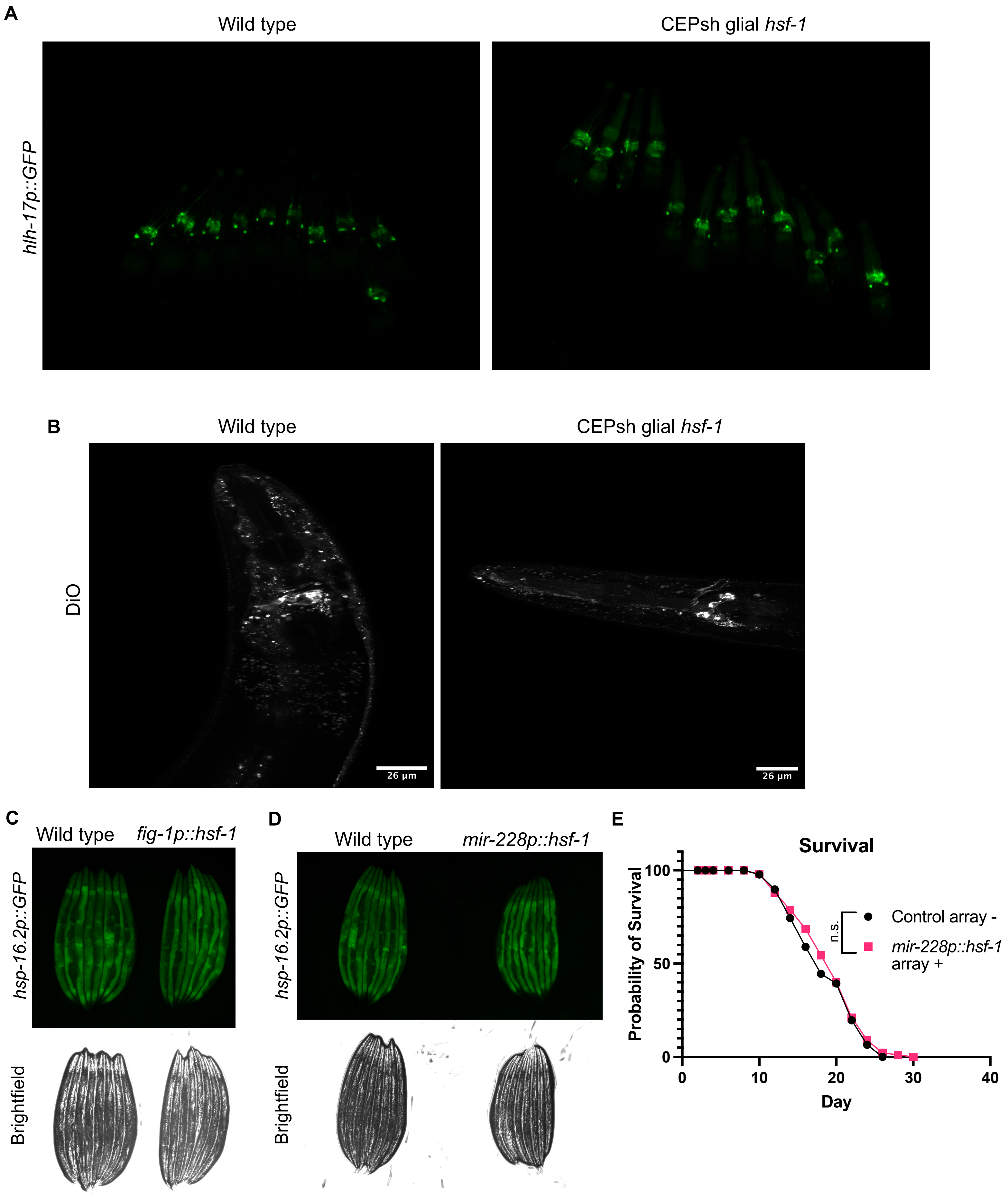

A
Wild type
CEPsh glial *hsf-1*
hlh-17p::GFP
B
Wild type
CEPsh glial *hsf-1*
DiO
26 µm
26 µm
C
Wild type
fig-1p::hsf-1
hsp-16.2p::GFP
Brightfield
D
Wild type
mir-228p::hsf-1
hsp-16.2p::GFP
Brightfield
E
Survival
Probability of Survival
100
50
0
0
10
20
30
40
Day
n.s.
Control array -
mir-228p::hsf-1 array +

S3

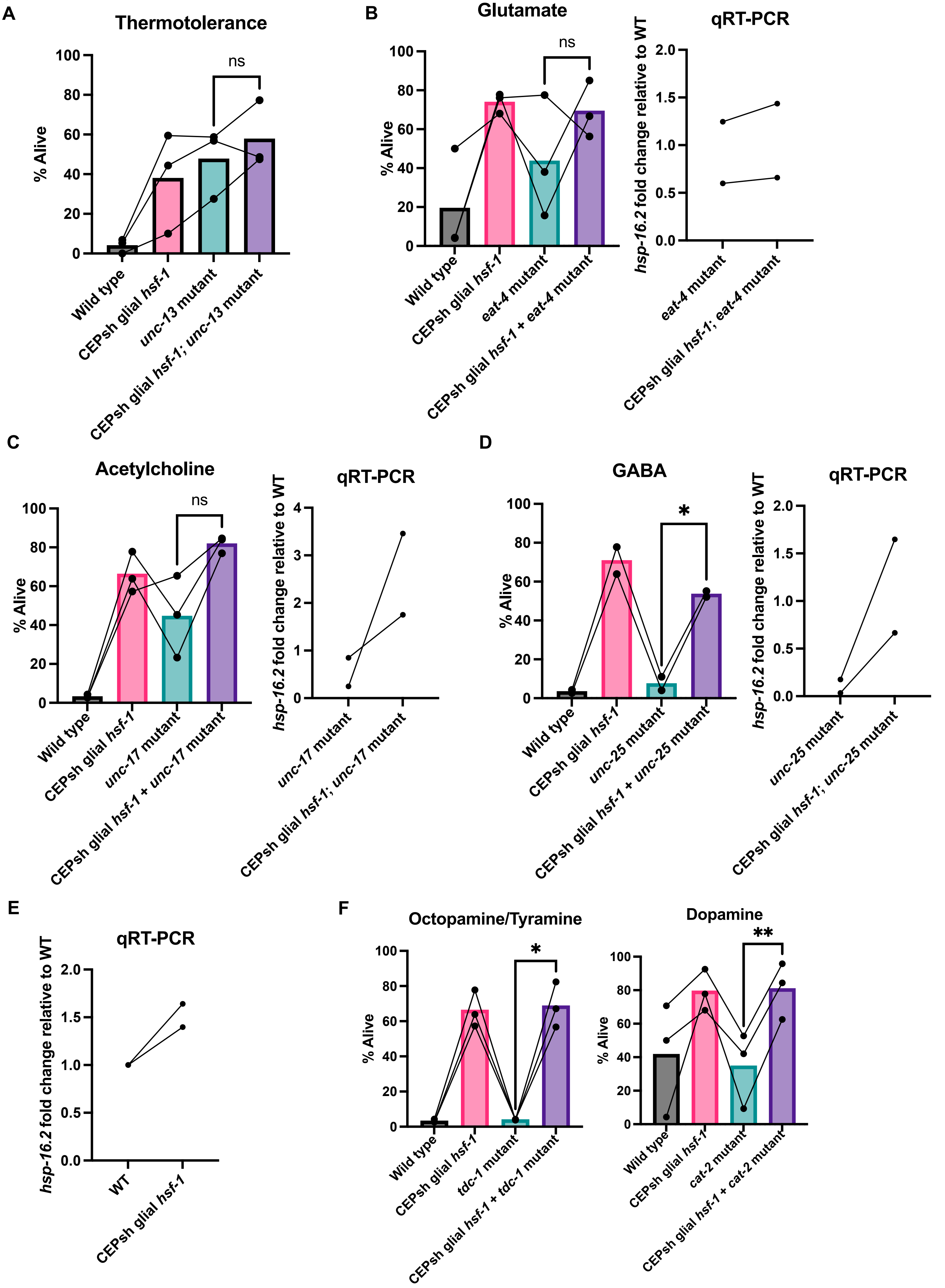
A
Thermotolerance
% Alive
100
80
60
40
20
0
ns
Wild type
CEPsh glial hsf-1
unc-13 mutant
CEPsh glial hsf-1; unc-13 mutant
B
Glutamate
% Alive
100
80
60
40
20
0
ns
Wild type
CEPsh glial hsf-1
eat-4 mutant
CEPsh glial hsf-1 + eat-4 mutant
qRT-PCR
hsp-16.2 fold change relative to WT
2.0
1.5
1.0
0.5
0.0
eat-4 mutant
CEPsh glial hsf-1; eat-4 mutant
C
Acetylcholine
% Alive
100
80
60
40
20
0
ns
Wild type
CEPsh glial hsf-1
unc-17 mutant
CEPsh glial hsf-1 + unc-17 mutant
qRT-PCR
hsp-16.2 fold change relative to WT
4
3
2
1
0
unc-17 mutant
CEPsh glial hsf-1; unc-17 mutant
D
GABA
% Alive
100
80
60
40
20
0
*
Wild type
CEPsh glial hsf-1
unc-25 mutant
CEPsh glial hsf-1 + unc-25 mutant
qRT-PCR
hsp-16.2 fold change relative to WT
2.0
1.5
1.0
0.5
0.0
unc-25 mutant
CEPsh glial hsf-1; unc-25 mutant
E
qRT-PCR
hsp-16.2 fold change relative to WT
2.0
1.5
1.0
0.5
0.0
WT
CEPsh glial hsf-1
F
Octopamine/Tyramine
% Alive
100
80
60
40
20
0
*
Wild type
CEPsh glial hsf-1
tdc-1 mutant
CEPsh glial hsf-1 + tdc-1 mutant
Dopamine
% Alive
100
80
60
40
20
0
**
Wild type
CEPsh glial hsf-1
cat-2 mutant
CEPsh glial hsf-1 + cat-2 mutant

S4

A

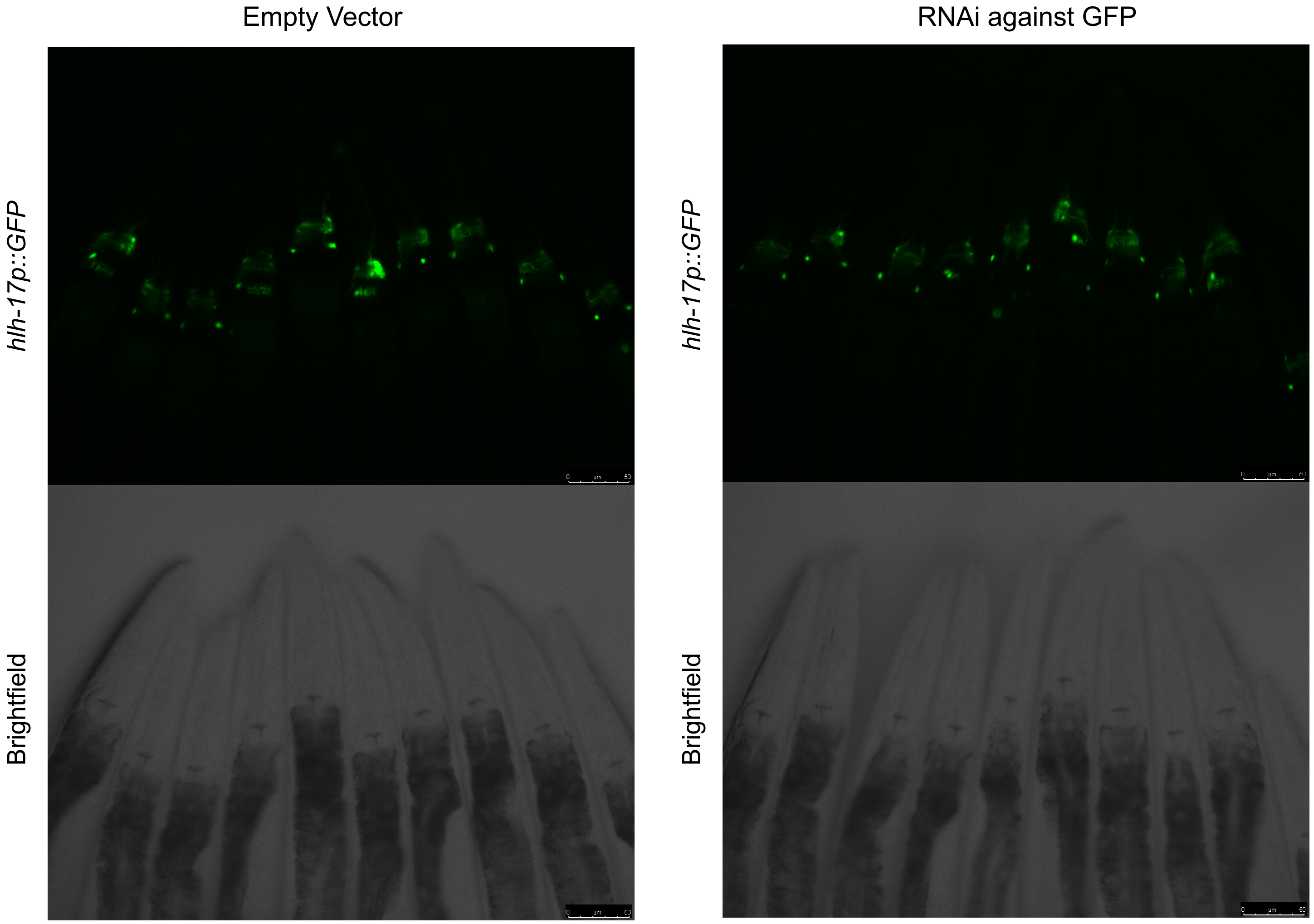
Empty Vector
RNAi against GFP
hlh-17p::GFP
Brightfield
hlh-17p::GFP
Brightfield

B

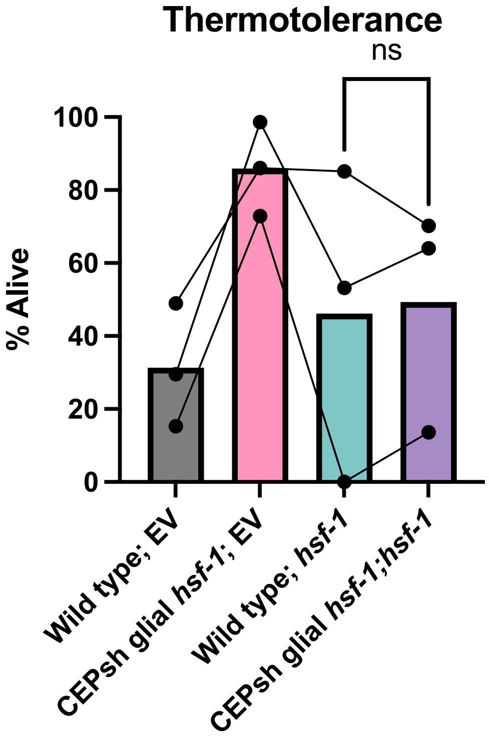
Thermotolerance
ns
% Alive
0
20
40
60
80
100
Wild type; EV
CEPsh glial hsf-1; EV
Wild type; hsf-1
CEPsh glial hsf-1;hsf-1

C

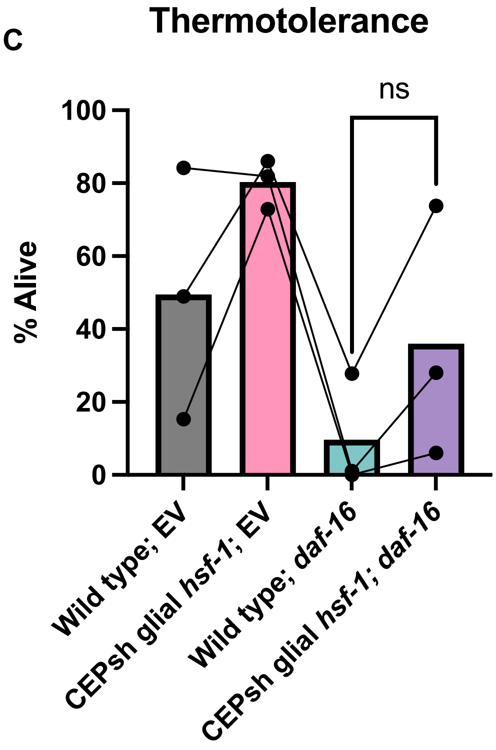
Thermotolerance
ns
% Alive
0
20
40
60
80
100
Wild type; EV
CEPsh glial hsf-1; EV
Wild type; daf-16
CEPsh glial hsf-1; daf-16

S5

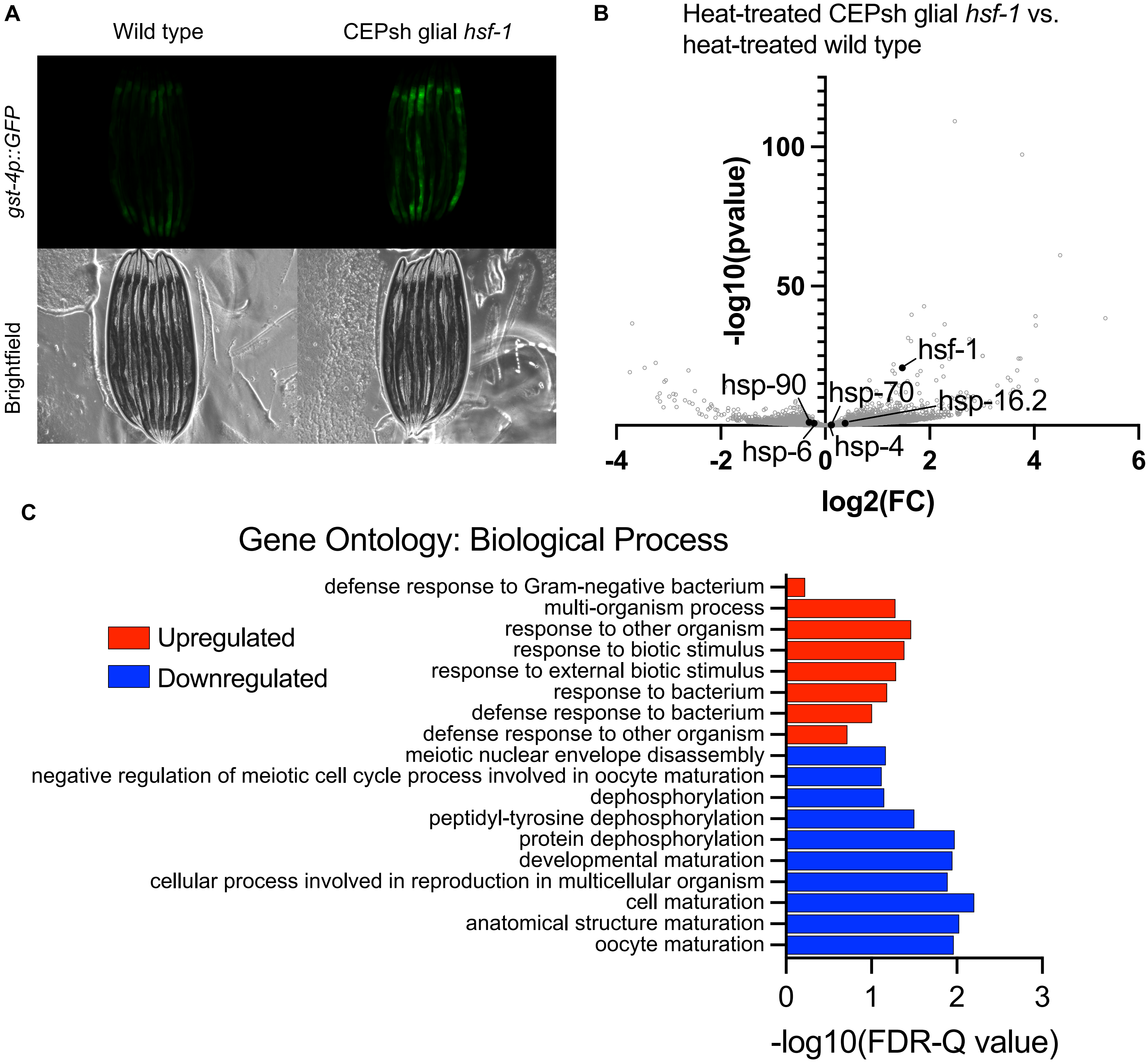
A
Wild type
CEPsh glial hsf-1
gst-4p::GFP
Brightfield
B
Heat-treated CEPsh glial hsf-1 vs.
heat-treated wild type
-log10(pvalue)
100
50
hsf-1
hsp-90
hsp-70
hsp-16.2
hsp-6
hsp-4
-4
-2
0
2
4
6
log2(FC)
C
Gene Ontology: Biological Process
Upregulated
Downregulated
defense response to Gram-negative bacterium
multi-organism process
response to other organism
response to biotic stimulus
response to external biotic stimulus
response to bacterium
defense response to bacterium
defense response to other organism
meiotic nuclear envelope disassembly
negative regulation of meiotic cell cycle process involved in oocyte maturation
dephosphorylation
peptidyl-tyrosine dephosphorylation
protein dephosphorylation
developmental maturation
cellular process involved in reproduction in multicellular organism
cell maturation
anatomical structure maturation
oocyte maturation
0
1
2
3
-log10(FDR-Q value)

Supplementary Figure 1: Phenotype of CEPsh glial *hsf-1* and reproductive effects A) Lifespan of independent integrated array *Is2(hlh-17p::hsf-1)* is increased relative to wild type N2 animals. Median of N2 = 21 days, median of *Is2(hlh-17p::hsf-1)* = 25 days, p < 0.001. B) Lifespan of extrachromosomal array *Ex(hlh-17p::hsf-1)* is increased relative to wild type N2 animals. Median of N2 = 17 days, median of *Ex(hlh-17p::hsf-1)* = 20 days, p < 0.0001. C) Total brood size over the reproductive lifespan; *Ex(hlh-17p::hsf-1)* have significantly fewer progeny than wild type N2, p < 0.001. Error bars are SD D) Brood size depicted across days of the reproductive lifespan. Error bars are SD E) Thermotolerance of independent integrated array *Is2(hlh-17p::hsf-1)* is increased relative to wild type N2 animals, p= 0.03. F) *hsp-16.2p::GFP* transcriptional reporter worms *Is2(hlh-17p::hsf-1)* versus wild type N2 after mild heat stress and recovery (left) and qRT-PCR of the same strains for *hsp-16.2* without heat stress without heat by standard curve analysis (right). G) *sod-3p::GFP* transcriptional reporter worms *Is2(hlh-17p::hsf-1)* versus wild type N2 under basal temperature conditions (left) and *hsp-70p::GFP* transcriptional reporter versus wild type N2 under heat stress (right), lined up head to tail. H) *hsp-4p::GFP* transcriptional reporter worms *Is2(hlh-17p::hsf-1)* versus wild type N2 (left), and *hsp-6p::GFP* transcriptional reporter worms *Is2(hlh-17p::hsf-1)* versus wild type N2 (right) under basal temperature conditions. Note that bleed-through from *myo-2p::tdtomato* in CEPsh glial *hsf-1* is visible at high exposure.

Supplementary Figure 2: Glial and neuronal health are maintained, and other glial subtype promoters are insufficient to activate a peripheral HSR A) *hlh-17p::GFP* fluorescent promoter imaging in wild type versus *Ex(hlh-17p::hsf-1)* animals demonstrates the morphology of CEPsh glia. CEPsh glial *hsf-1* animals maintain neuropil wrapping and gross process extension, but sometimes display loss of symmetry. B) DiO-based dye filling of neurons in wild type versus *Ex(hlh-17p::hsf-1)* animals demonstrates normal presence of neurons. C) *Ex(fig-1p::hsf-1); hsp-16.2p::GFP* animals (right) do not display consistent increase in fluorescence relative to *hsp-16.2p::GFP* alone (left) D) *Ex(mir-228p::hsf-1); hsp-16.2p::GFP* animals (right) do not display consistent increase in fluorescence relative to *hsp-16.2p::GFP* alone (left) E) Survival of *mir-228p::hsf-1* (extrachromosomal array) is not significantly increased relative to array negative animals at 20°C.

Supplementary Figure 3: Neurotransmitter requirements for signaling A) Thermotolerance of *Ex(hlh-17p::hsf-1); unc-13(s69)* is not significantly increased relative to *unc-13(s69)* alone, although inter-trial variability is high. B-D) Thermotolerance and qRT-PCR of small clear vesicle-transported neurotransmitter mutants. Mutants for each neurotransmitter alone are displayed in blue, and CEPsh glial *hsf-1* with the relevant neurotransmitter mutant is displayed in purple. B) Thermotolerance of *Ex(hlh-17p::hsf-1); eat-4(ky5)* is not significantly increased relative to *eat-4(ky5)* alone, though there is a trend towards increase (left). Levels of *hsp-16.2* are mildly increased by qRT-PCR (right). C) Thermotolerance of *Ex(hlh-17p::hsf-1); unc-17(e245)* is not significantly increased relative to *unc-17(e245)* alone, though there is a trend towards increase (left). Levels of *hsp-16.2* are increased by qRT-PCR (right). D) Thermotolerance of *Ex(hlh-17p::hsf-1); unc-25(e156)* is significantly increased relative to *unc-25(e156)* alone, p=0.02 (left). Levels of *hsp-16.2* are increased by qRT-PCR (right). E) qRT-PCR of *Ex(hlh-17p::hsf-1)* animals alone, displaying wild type N2 conditions to which displayed trials are normalized. F) Thermotolerance of presumed dense core vesicle-transported neurotransmitter mutants. Mutants for each neurotransmitter alone are displayed in blue, and CEPsh glial *hsf-1*

with the relevant neurotransmitter mutant is displayed in purple. Thermotolerance of *Ex(hlh-17p::hsf-1); cat-2(n4547)* is significantly increased relative to *cat-2(n4547)* alone, p= 0.03 (right). Thermotolerance of *Ex(hlh-17p::hsf-1); tdc-1(n3419)* is significantly increased relative to *tdc-1(n3419)* alone, p= 0.04 (left).

Supplementary Figure 4: Effects of RNAis on CEPsh glia A) CEPsh glial GFP via *hlh-17p::GFP* is not knocked down when exposed to RNAi against GFP (right), as compared to signal on empty vector bacteria (left) B) Thermotolerance of *Ex(hlh-17p::hsf-1)* eating *hsf-1* RNAi bacteria (blue) is not significantly increased relative to that of wild type N2 worms eating *hsf-1* RNAi (purple). C) Thermotolerance of *Ex(hlh-17p::hsf-1)* eating *daf-16* RNAi bacteria (blue) is not significantly increased relative to that of wild type N2 worms eating *daf-16* RNAi (purple).

Supplementary Figure 5: Validation of RNA sequencing and heat-shocked RNA sequencing A) CEPsh glial *hsf-1* animals display increased *gst-4p::GFP* relative to wild type animals. B) Volcano plot demonstrating magnitude (Log2(FC)) and significance (-log10(p-value)) of changes in gene expression from whole-animal RNA sequencing of *Is1(hlh-17p::hsf-1)* versus wild type N2 after a 30 minute, 34ºC heat shock. Labeled genes are stress genes, including *hsf-1* and HSR chaperones *hsp-70*, *hsp-16.2* (HSF-1 regulated) and *hsp-90* (non-HSF-1 regulated), as well as ER UPR chaperone *hsp-4* and mitochondrial UPR chaperone *hsp-6*. C) The top Gene Ontology (GO) terms for up- (red) and down- (blue) regulated genes in *Is1(hlh-17p::hsf-1)* versus wild type N2 after a 30 minute, 34ºC heat shock are displayed with their -log10 corrected FDR-Q value.
